# A practical protocol for correlative confocal fluorescence and transmission electron microscopy characterization of extracellular vesicles

**DOI:** 10.1128/spectrum.03026-24

**Published:** 2025-05-22

**Authors:** Juan M. Martínez-Andrade, Daniel Salgado-Bautista, Kendra Ramirez-Acosta, Ruben Darío Cadena-Nava, Meritxell Riquelme

**Affiliations:** 1 Department of Microbiology, Centro de Investigación Científica y Educación Superior de Ensenada (CICESE) 7082, Ensenada, Baja California, Mexico; 2 Centro de Nanociencias y Nanotecnología, Universidad Nacional Autónoma de México 87793, Ensenada, Baja California, Mexico; CNRS-Inserm-Université Côte d'Azur, Nice, France

**Keywords:** extracellular vesicles, correlative light and electron microscopy, transmission electron microscopy, laser scanning confocal microscopy

## Abstract

Extracellular vesicles (EVs) have gained considerable attention in fungal biology research. However, imaging these membrane-bound particles presents challenges due to their nanoscopic size (typically <200 nm), which exceeds the resolution limit of conventional diffraction-limited laser scanning confocal microscopy (LSCM). While high-resolution techniques like transmission electron microscopy (TEM) offer superior spatial resolution, they are time-consuming, require specialized expertise, and are prone to artifacts that can interfere with accurate results. In this study, we propose a rapid method for confirming the vesicular nature of EVs using a correlative light and electron microscopy (CLEM) approach. EVs were isolated from culture filtrates of the model filamentous fungus *Neurospora crassa,* and their membranes were stained with the fluorogenic styryl dye FM1-43 for analysis. Fluorescent microspheres were used as fiducial markers alongside the stained EVs during sample preparation. Samples were first examined using LSCM, followed by negative staining with OsO_4_ vapors or uranyl acetate. The same regions observed with LSCM were subsequently analyzed with TEM. CLEM analysis revealed that vesicle-like structures with membranous features, as observed under TEM, corresponded to the dispersed green fluorescence signal seen with LSCM. These findings validate CLEM as a reliable method to examine the presence of EVs. This method can be implemented easily in labs that do not have access to a core facility with sophisticated multimodal microscopes. Furthermore, this method can be extended to study other structures, such as secretory vesicles, viral particles, protein nanoparticles, and polymeric nanoparticles.

**IMPORTANCE:**

This study presents an efficient and cost-effective correlative light and electron microscopy workflow for imaging nanosized extracellular vesicles (EVs) and other biological samples. The methodology involves sequential imaging using laser scanning confocal microscopy (LSCM) followed by transmission electron microscopy (TEM), enabling comprehensive characterization of EVs. This protocol uses fluorescence microscopy dyes to stain EV membranes and OsO_4_ vapors for negative or positive staining in TEM. This approach provides a reliable, versatile tool for studying nanoscale biological structures, with broad applications in cellular biology, nanomedicine, and related research fields.

## INTRODUCTION

Extracellular vesicles (EVs) are quasi-spherical structures surrounded by a double lipid membrane, produced by all cell types. Based on their mode of release into the extracellular space, EVs are classified into apoptotic bodies, microvesicles (also known as ectosomes), and exosomes (1, 2). Exosomes typically range from 30 to 150 nm, ectosomes from 50 to 1,000 nm, and apoptotic bodies can be as large as 5,000 nm (1, 3, 4).

In bacterial and fungal cells, EVs play crucial roles in antibiotic and antifungal resistance, as well as in virulence (5, 6). Recently, there has been growing interest in the potential of EVs to carry antibiotics for targeted delivery to inaccessible sites (5) and in the immunomodulatory capabilities of fungal EVs for vaccine development (6, 7).

To analyze isolated EVs, techniques such as OMICS, nanoparticle tracking analysis, and dynamic light scattering are commonly employed to characterize their composition and size (8). However, these methods often must be complemented with light and electron microscopy to confirm the morphology of the EVs. The choice of microscopy technique depends on whether the study is conducted *in situ* or *in vitro*, each with its inherent limitations.

Conventional fluorescence microscopy (FM) techniques have provided valuable insights regarding the release, uptake, and distribution of EVs in live cells (9). Labeling is required to visualize EVs through genetic modifications or vital dyes. However, only EVs larger than ≥250 nm are easily detected with diffraction-limited microscopy approaches, making it challenging to visualize smaller EVs (<250 nm).

In contrast, electron microscopy (EM) offers exceptional spatial resolution (<1 nm), allowing for detailed characterization of EVs’ shape and size particle distribution (10). Transmission electron microscopy (TEM), often combined with negative staining, is widely used to evaluate the morphology and size of EVs (11). However, the preparation of biological samples for EM involving fixation, post-fixation, dehydration, and negative staining can introduce artifacts such as impurities. These artifacts can complicate the distinction between the sample and background noise. Some studies have explored using algorithms to differentiate EVs from other artifacts (11).

Combining FM and EM allows researchers to overcome the resolution limits of optical microscopy by using fluorescent signals to provide detailed context at the ultrastructural level. This technique, known as correlative light and electron microscopy (CLEM), offers various implementation methods, including integrated “all-in-one” microscopy systems. However, access to these advanced systems is limited. A range of CLEM protocols have been developed over the years, combining traditional and super-resolution light microscopy with TEM, scanning electron microscopy (SEM), focused ion beam scanning electron microscopy, and even cryo-electron microscopy, which allows visualization of biological systems in a near-native state (12). Some of these methods require additional steps or specialized, often costly, equipment to handle and visualize samples. As a result, the complexity of the technique and the level of skill needed can vary significantly depending on the specific CLEM method used.

Recently, several CLEM protocols have been developed to track EVs and to localize molecular markers in EVs (13). Most of these protocols combine FM with SEM and investigate EVs larger than 100 nm. For example, CLEM using SEM was used to image EVs released from human mesenchymal stem cells (14) and the interaction of EVs with recipient cells (15). However, only a few studies have explored the combination of FM with TEM. For example, this approach was employed to track labeled tumor EVs (>50 nm in size) in zebrafish embryos (9) and to localize CD63 and CD9 in isolated EVs (≤100 nm in size) from malignant brain tumors (16). To date, there are no reports of CLEM applications for fungal or bacterial EVs.

We have developed and tested a straightforward technique for performing CLEM using laser scanning confocal microscopy (LSCM) and TEM to study EVs isolated from the model filamentous fungus *Neurospora crassa*. Our approach involves using a lipophilic dye from the FM family to rapidly localize membranous structures, as these dyes emit fluorescence only when intercalating into the lipid bilayer (17, 18). Subsequently, the samples are evaluated using TEM, with osmium tetroxide (OsO_4_) vapors used to stain EVs, minimizing sample movement. This method offers significant advantages over traditional negative staining with liquid solutions such as uranyl acetate, by reducing the risk of introducing extrinsic particles that may resemble EVs and lead to artifacts (19). Additionally, the method is cost-effective, requiring neither expensive equipment nor complex tools to combine the two microscopy modalities. Furthermore, this technique can be applied to study EVs with unknown cargo or used as a screening method to differentiate between artifacts and authentic EV samples. This technique can also be integrated with algorithm-based super-resolution imaging, diaminobenzidine photo-oxidation using FM1-43 (20, 21), or genetically encoded fluorescent tags.

## MATERIALS AND METHODS

### EVs isolation

The EVs used in this work were isolated from *N. crassa* culture filtrates as previously described (19). *N. crassa* strain FSGC #988 was grown in 200 mL of Vogel’s minimal medium with 1.5% sucrose as the carbon source in a 500 mL Erlenmeyer flask. The growth conditions included 48 h at 30°C, 150 rpm in the absence of light. The fungal biomass was separated by filtration using Whatman #1 paper. The recovery of EVs was carried out using differential centrifugation. To remove cell debris and apoptotic bodies, the filtrate was centrifuged sequentially at 3,000 rpm for 30 min and at 10,000 rpm for 1 h. The final supernatant was ultracentrifuged (Optima MAX-XP Beckman Coulter) at 100,000 × *g* (using an MLA-50 fixed-angle rotor) for 2 h. The resulting pellet was resuspended in 200 µL of 20 mM Tris-HCl (pH 7.2) and centrifuged at 10,000 × *g* for 10 min to eliminate agglomerated particles. The supernatant was then recovered and diluted 1:1 with 20 mM Tris-HCl (pH 7.2) for further analysis.

### Purification of brome mosaic virus

Brome mosaic virus (BMV) virions were purified from infected barley leaves, as described by Ramirez-Acosta et al. (22). Two weeks after germination, barley leaves were mechanically damaged. Then, 10 µL of a virus suspension (0.1 mg/mL) was gently rubbed onto the damaged site. Two weeks later, leaves showing symptoms of infection were harvested and blended with 2 mL of solution A (500 mM NaOAc, 80 mM MgOAc, 1 mM 2-mercaptoethanol, pH 7.4) per gram of leaves. The mixture was then filtered through cheesecloth. Chilled chloroform was added to the filtrate, and the solution was stirred for 10 min at 4°C. In order to separate the plant material, the solution was first centrifuged at 15,000 × *g* for 20 min at 4°C. The aqueous phase was then collected and subjected to a second centrifugation at 15,000 × *g* for 15 min at 4°C. The resulting supernatant was collected, and NaCl and PEG-8000 were added to achieve final concentrations of 200 mM and 10% (w/v), respectively. The mixture was stirred for 1 h at 4°C before being centrifuged at 10,000 × *g* for 20 min at 4°C. After centrifugation, the supernatant was discarded. The pellet was resuspended in 75 mL of sodium acetate, magnesium acetate (SAMA) buffer (50 mM NaOAc, 8 mM NaOAc, pH 4.5) and ultracentrifuged at 126,000 × *g* for 150 min at 4°C, using a 10% sucrose cushion. The final pellet was resuspended in SAMA buffer, and virus concentration was determined by measuring the UV-Vis absorbance at 260 and 280 nm using a Nanodrop 2000c spectrophotometer (ThermoFisher Scientific). The purified virus was aliquoted into 1.5 mL microcentrifuge tubes and stored at −80°C.

### Brome mosaic virus-fluorescein-5-isothiocyanate

The external surface of BMV virions was labeled with fluorescein-5-isothiocyanate (FITC) through covalent conjugation to the solvent-exposed amino groups as previously described (23, 24). Specifically, 300 µg of BMV (65.2 pmol) was labeled with a 200-fold molar excess of FITC per viral subunit in phosphate buffer saline (PBS), under constant agitation for 2 h at room temperature. Excess fluorophore was removed using 100 kDa Amicon ultrafiltration filters (0.5 mL, Millipore).

### Laser scanning confocal microscopy

A mixed solution was made with the isolated EVs diluted with FM1-43 (SynaptoGreen C4, Cat# 70020, Biotium) to achieve a dye concentration of 5 µM. Then, 1 µL of fluorescent polystyrene microspheres (Fluoresbrite polychromatic red [PC-RED] microspheres, 1 µm in diameter; Polysciences, Inc., no. 1866, 2.5% wt/vol) was added to 99 µL of EVs + FM1-43 solution to obtain a 1:100 dilution of the fiducial markers in the final solution. In addition, fluorescent nanobeads of 160 nm (Dye XC, concentration 1%, Estapor Microspheres) and 100 nm (TetraSpeck, ThermoFisher) were employed for the complementary analysis for negative staining with uranyl acetate.

A triplicate of samples was prepared from the final solution for CLEM analysis. Ten microliters of the sample was deposited onto each 75-mesh formvar-carbon copper grid (Ted Pella, Inc.) and allowed to stand for 1 min. After that, the excess liquid was removed with blotting paper. The copper grids were placed on a glass coverslip (VWR no. 1, 21 × 26 mm) and covered with another glass coverslip (VWR, 99.1 × 33.9 mm).

The grids were examined using an inverted Olympus Fluoview FV1000 laser scanning confocal microscope fitted with an argon laser for FM1-43 and nanobeads (TetraSpeck) visualization (excitation: 488 nm, emission: 505–525 nm) and a diode-pumped solid-state (DPSS, Melles Griot, Carlsbad, CA, USA) laser for PC-RED microspheres visualization (excitation: 543 nm, emission: 560 nm).

For CLEM analysis, EVs were imaged using 20× (UPlanSapo, 0.75 N.A.), 40× (UPlanFL, 1.30 N.A. Oil), and 60× (PlanApo N/A.N. 1.42 Oil) objectives. Confocal images were captured at a resolution of 1,024 × 1,024 pixels, with a scanning speed of 12.5 µm/s and a pinhole size of 100 µm. Image analysis was performed using FV10-ASW software (version 4.0.2.9, Olympus). The grids were examined quadrant by quadrant, starting from the center grid, which was used as the initial coordinate reference.

### Transmission electron microscopy

For negative staining, two methods were compared:

1. OsO_4_ vapors: osmium tetroxide crystals were dissolved in an aqueous solution at a concentration of 2% (wt/vol) prior to use. The grids were held with tweezers 2–5 cm above the lids of 1.5 mL Eppendorf tubes containing OsO_4_ solution for at least 10 min inside a fume hood, following the procedure previously described (19). Depending on the sample type and concentration, a staining time of 5 min may be sufficient. However, for certain samples, such as the BMV virus, the staining times were extended to 15–30 min, and they were treated with OsO_4_, which had been previously prepared in acetone, to enhance the contrast of the vapors. In addition, EVs stained with FM1-43 were exposed to varying times of OsO_4_ vapors to determine whether the coordinates of fiducial markers were altered and how the fluorescence signal changed over time.
2. Uranyl acetate: 2% uranyl acetate (wt/vol) was prepared in aqueous form and centrifuged at 11,000 rpm for 10 min before use. After LSCM observations, 10 µL of the uranyl acetate solution placed onto the grid, allowed to settle for 60 s, and then gently blotted off. A control group of grids containing fluorescent beads and stained with uranyl acetate was evaluated before and after blotting to determine if the coordinates of the fluorescent beads were altered.

Finally, grids loaded with fluorescent beads but without EV samples were evaluated in TEM before and after staining with OsO_4_ vapors and uranyl acetate to assess the presence of artifacts.

Observations were conducted at both low magnification (100×–700×) and high magnification (20,000×–50,000×) using a Hitachi H7500 transmission electron microscope (thermionic emission, 80 kV) equipped with a 16-megapixel Gatan CCD digital camera. The software was calibrated with a scale bar generated from TEM images using a reference sample grid containing polystyrene particles 100–500 nm in diameter.

### Imaging processing and correlative analysis

Objectives of 20×, 40×, and 60× in LSCM and 100× magnifications in TEM were used to correlate the positions of the fluorescent microspheres. The regions of interest (ROIs) from this initial map were defined and explored at higher magnifications (20,000×–50,000×) using TEM. The micrographs were processed with Fiji’s open-access software (version 2.14.0/1.54f). The particle diameter (*n* = 100) was obtained from ROIs of the different fields selected in the micrographs. The alignment of LSCM and TEM micrographs was conducted in Adobe Photoshop CS6 version 13.0. At higher magnification in TEM, more than three beads were considered to ensure precise alignment; however, other landmarks, such as the patterns of the sample location, were also used for the alignment.

Nanoscopic images were obtained using Fiji’s mean-shift super-resolution (MSSR) plugin (25). To this end, an amplification parameter value of 3 and an order analysis value of 1 were employed. The point spread function value was obtained using the “ImageDecorrelationAnalysis” plugin.

### Statistical analysis

The average, standard deviation, and frequency distribution were obtained to calculate the size of EVs. GraphPad Prism version 6.0c was used for this purpose.

## RESULTS AND DISCUSSION

### Easy and quick CLEM workflow confirms the presence of membranous EVs

**Fig 1:**
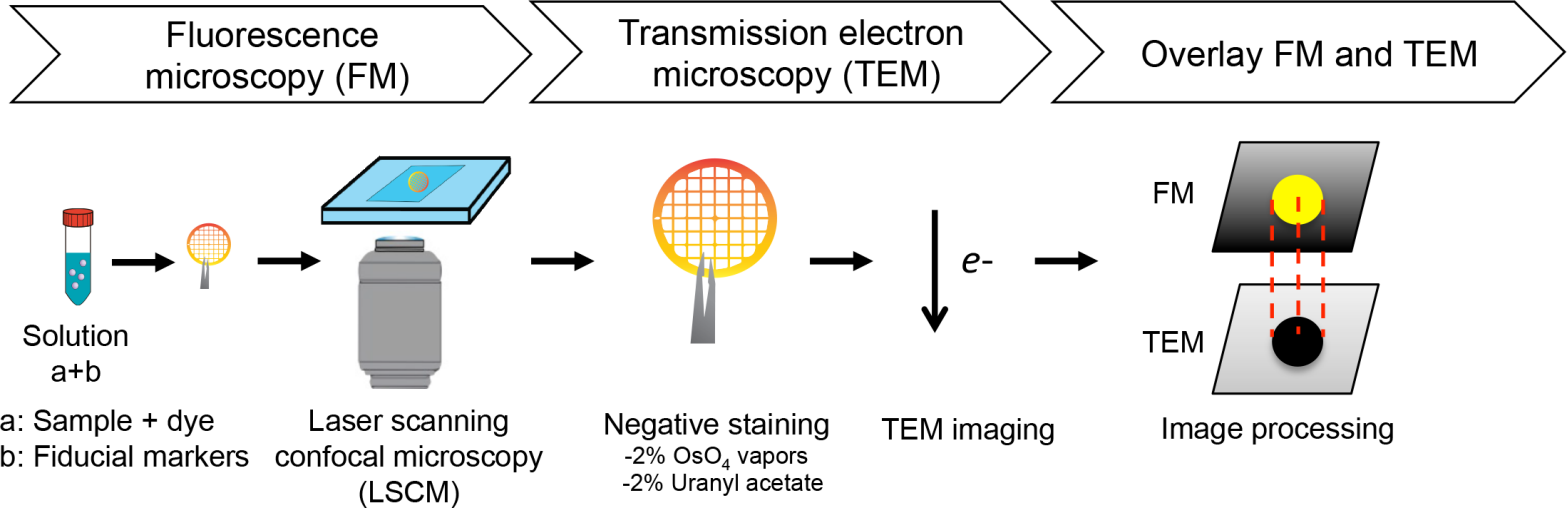
Workflow for CLEM analysis of EVs using LSCM and TEM. The isolated EVs are stained with FM1-43, mixed with fluorescent microspheres as fiducial markers, and deposited onto a formvar-carbon copper grid. Then, the grid is observed at low magnification using LSCM (i.e., 20×–40×), and the ROI is selected. The grid is exposed to 2% OsO_4_ vapors for a short duration (5–10 min) or 2% uranyl acetate (60 s). The sample is visualized at low (100×–700×) and high magnifications (20×–50,000×) by TEM. Finally, LSCM and TEM images are overlaid using Adobe Photoshop, considering ROIs and the fiducial markers or any other landmarks as coordinates.

In this study, we developed a user-friendly CLEM workflow for imaging EVs recovered from *N. crassa* culture filtrates (Fig. 1). This method involves five steps that combine the use of a membrane-specific fluorescent dye (FM1-43) with negative staining using OsO_4_ vapors, enabling sequential imaging of EVs via LSCM and TEM. Fluorescent patterns in FM1-43-stained samples were observed with low magnification objectives (e.g., 20×–40×) in LSCM. Low magnifications in both LSCM and TEM provided a wide field of view of the 75-mesh grid squares, facilitating precise alignment of fluorescence and electron microscopy images and the identification of fiducial markers and landmarks such as grid edges.

**Fig 2:**
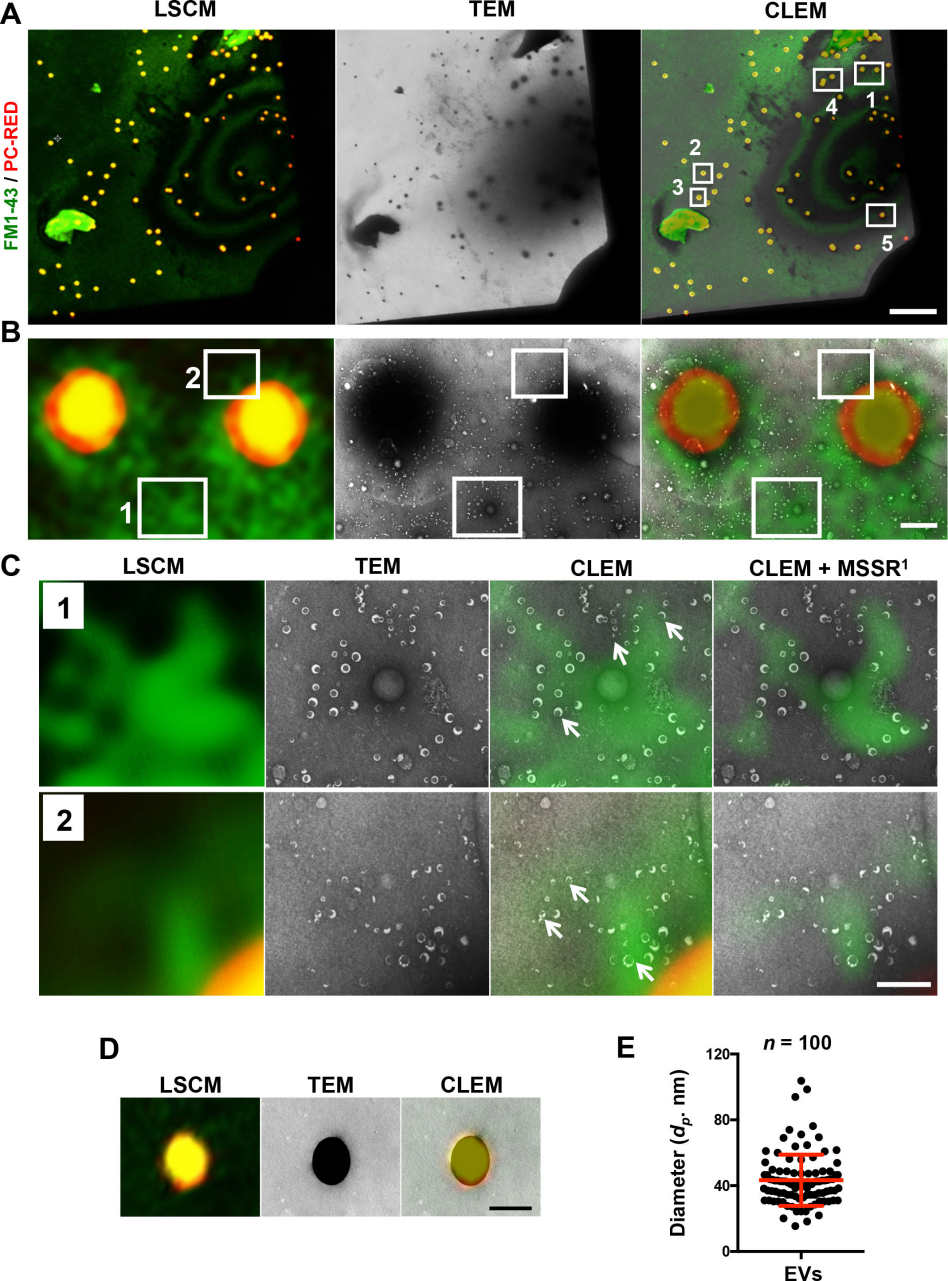
LSCM and TEM micrographs of EVs stained with 2% OsO_4_ vapors for CLEM analysis. (**A**) Micrographs of LSCM and TEM show the general map of the sample at low magnification. Five ROIs were selected based on the CLEM (white squares). (**B**) ROI 5 with high (square 1) and low (square 2) green fluorescence signal areas were analyzed at high magnification. (**C**) Insets of the selected areas in (B) show the green fluorescence signal with CLEM and CLEM + MSSR. Arrows indicate different sizes of EVs. (**D**) ROI 2 selected as negative control. (**E**) The diameter of the EVs (*n* = 100) was obtained from the different selected ROIs. Scale bars: (A) 25 µm, (B) 1 µm, (C) 0.5 µm, and (D) 1 µm.

To minimize correlation steps, we employed low magnifications from the outset and introduced 1 µm fluorescent PC-RED microspheres, which are readily visible in both light and electron microscopy (Fig. 2A). Notably, we found a higher concentration of EVs near these fluorescent microspheres (Fig. 2B), enhancing the correlation between the fluorescent signal and the ultrastructural details. PC-RED microspheres are internally dyed with multiple fluorophores (R-PE, Rhodamine, Nile Red, CyTM3, Alexa Fluor 532, Alexa Fluor 546, and SYTOX Orange), offering broad excitation bands, and they allow either covalent coupling or passive adsorption of proteins to their surface. This interaction likely accounts for the clustering of EVs observed near the microspheres.

OsO_4_ vapors, previously shown to be an effective alternative to uranyl acetate for TEM imaging of EVs (19), offer the advantage of a “free-touch” staining method. This preserves the sample’s original positioning during analysis across different microscopy modalities while maintaining fiducial markers intact. Additionally, OsO_4_ vapors provide some fixation for EVs, although the extent depends on exposure duration. Despite some EV damage observed (Fig. 2C), likely due to high vacuum conditions and residual water in the sample, we opted to omit dehydration and fixation steps to streamline the workflow. Although chemical fixation, cryofixation, and alcohol dehydration could enhance preservation, their inclusion depends on specific experimental needs (11).

### Correlative analysis of fluorescence and ultrastructural features

We selected five ROIs based on fluorescence patterns and contrast observed in the overlaid LCSM and TEM images (Fig. 2A). ROI 1 revealed abundant quasi-spherical structures resembling EVs in TEM images that correlated with high-intensity green fluorescence signal near fiducial markers (Fig. 2B and C1). Areas with weaker fluorescence signals contained fewer EVs (Fig. 2B and C2). Enhanced spatial resolution using MSSR further improved the overlay of FM1-43 fluorescence signals with vesicle-like structures; however, other EVs were observed without a signal (Fig. 2C). Negative control regions (ROIs 2 and 3) with minimal green fluorescence around the fiducial marker showed no EV-related electron microscopy contrast (Fig. 2D; see Fig. S1A at https://doi.org/10.6084/m9.figshare.28800383.v4). Similarly, ROIs 4 and 5 showed correlations between fluorescent intensity and EV distribution near microspheres in TEM images (see Fig. S1A at https://doi.org/10.6084/m9.figshare.28800383.v4). This correlation was further validated by higher magnification reanalysis (see Fig. S1B and C at https://doi.org/10.6084/m9.figshare.28800383.v4).

FM1-43 successfully stained membranous vesicle-like particles, with an estimated average diameter of 43.32 ± 15.56 nm (Fig. 2E), consistent with previously reported sizes of *N. crassa* EVs (19). Traditionally used in studies of neural cells and fungi (17, 26), FM1-43 has a higher membrane dissociation constant than FM4-64, FM2-10, and FM5-95 (18). The dye features a lipophilic tail and a highly hydrophilic, cationic head group that integrates into the lipid bilayer, where it emits fluorescence (27). Other studies have used FM1-43 to demonstrate EVs presence, but without utilizing CLEM, they have not been able to correlate fluorescence with EVs ultrastructure. For instance, FM1-43 combined with flow cytometry recently confirmed the presence of EVs from *Lactococcus cremoris* in bulk (28). Other FM dyes, such as FM4-64, have been employed to study EVs from *Lactiplantibacillus* species using fluorescence spectrometry (29) and to image EVs from *Ustilago maydis* at low resolution (30). In our study, higher fluorescence signal of FM1-43 coincided with areas where more EVs were identified, although in some areas individual EVs did not show fluorescence. One possible explanation for the lack of consistent staining of individual EVs with FM1-43 is that EVs tend to aggregate rather than remain separate, and the spatial resolution of confocal fluorescent imaging is a limiting factor to see individual EVs. It is possible that the FM1-43 signal could be detected in multiple EVs at once. Although we used nanoscopic deconvolution analysis to enhance spatial resolution, this technique is limited to spaces no smaller than 30 nm and can discriminate weak fluorescence signals in the sample (25), which may also account for the absence of discrete signals in some EVs and/or for the lack of fluorescent signal completely in other EVs.

**Fig 3:**
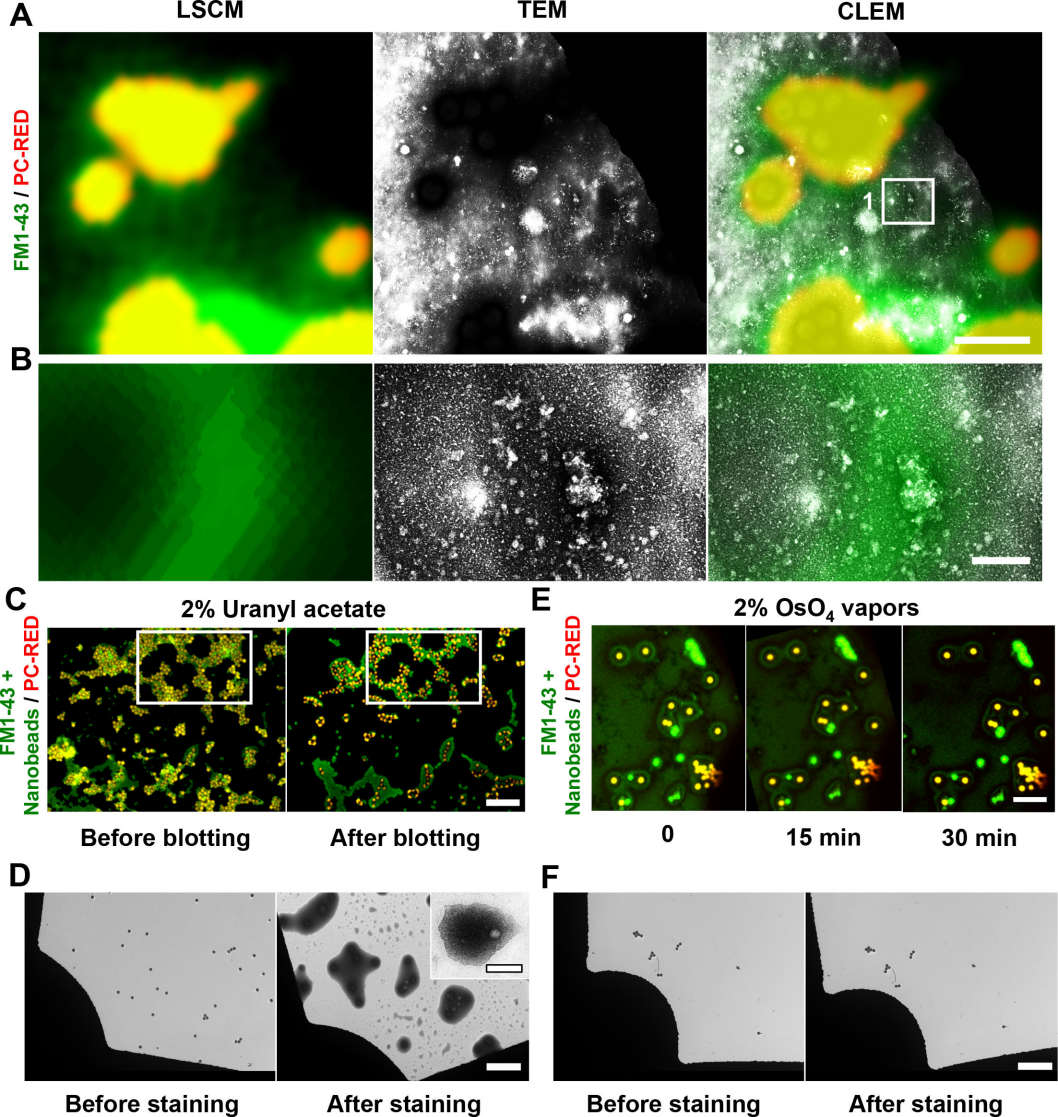
LSCM and TEM micrographs of EVs stained with 2% uranyl acetate for CLEM analysis. (**A**) LSCM and TEM micrographs showing the general map of the sample at low magnification. An ROI was selected based on the CLEM (white square). (**B**) The ROI was analyzed at high magnification. (**C**) Areas selected before and after blotting and (D) stained with 2% uranyl acetate. The white rectangle indicates the same reference point during the processes. (**D**) TEM assessment before and after uranyl acetate staining, with an inset showing a quasi-spherical structure post-staining. (**E**) Areas selected during the OsO_4_ vapors staining process at different times. (**F**) TEM assessment before and after OsO_4_ vapors staining. Scale bars: (A) 2 µm, (B) 0.2 µm, (C–F) 10 µm, and inset in (D) 0.5 µm.

We applied our CLEM method to EVs stained with 2% uranyl acetate. The CLEM analysis revealed a similar green signal overlapping quasi-spherical structures with an average size of 37.20 ± 13.12 nm (Fig. 3A and B). However, some fluorescent beads moved after the blotting step in the uranyl acetate-stained sample (Fig. 3C). Additionally, we observed particle-like artifacts on the grid without a sample, only stained with uranyl acetate (Fig. 3D). In contrast, the fluorescent beads maintained the same position at different intervals when using OsO_4_ vapors, and no dirty fields or extrinsic particles were observed (Fig. 3E and F).

We employed FITC-labeled BMV particles as an experimental control to ensure that our CLEM workflow works. To this end, an ROI was selected at the center of the grid, with nearby bars serving as reference points to facilitate locating the sample under LSCM and TEM (see Fig. S2A at https://doi.org/10.6084/m9.figshare.28800383.v4). CLEM analysis revealed a consistent pattern of sample distribution in both FM and EM modalities, including particle agglomeration. The presence of viral capsids was further confirmed at higher TEM magnification (see Fig. S2, B1-3.1 https://doi.org/10.6084/m9.figshare.28800383.v4).

One of the primary challenges in CLEM is to be able to accurately correlate the fluorescent signal from FM with corresponding features in EM. Tracing specific fluorescent cells or subcellular structures within a large data set requires easily identifiable reference points in both modalities. Previous CLEM protocols have utilized internal features, such as grid bars, edges, or characters, as well as external particles, including random contaminants, to manually correlate grid squares or areas of interest. Additionally, intrinsic cellular features, such as the ultrastructure of biological samples, have served as fiducial markers (31–33).

Several Cryo-CLEM studies have employed strategies similar to our CLEM protocol. For example, a combination of low (i.e., 100×–200×) and high magnifications (i.e., 6,000×–40,000×) in EM images, aligned with FM images, has been used to identify suitable ROIs for locating herpes simplex virus capsids and HIV-1 particles (31, 34). This approach has also been applied to localize actin-rich membranous protrusions in cells (32).

To our knowledge, only one study has combined negative staining and FM for CLEM in EV research (16). That study allowed the localization of CD63 and CD9 in EVs from mammalian cells using fluorophore-conjugated antibodies. After LSCM, the grids were negatively stained with 1% uranyl acetate in water and examined using TEM. The overlay of the fluorescent signals with TEM images revealed the localization of a limited number of EVs. However, this method also introduced artifacts likely due to the negative staining process. Additionally, the need to blot excess water after applying uranyl acetate may compromise the preservation of the fiducial markers.

### Conclusion

This study presents an efficient and cost-effective CLEM workflow for imaging EVs and other spherical particles. This protocol is versatile, adaptable to diverse sample types, and minimizes sample disruption by combining negative or positive staining with FM dyes. Future improvements for enhanced accuracy could include using microspheres of different sizes, around 200 nm, that are distinguishable in both imaging modalities. Furthermore, while OsO_4_ is hazardous, utilizing commercially available pre-prepared 2% solutions in volumes of 2–5 mL can optimize its use, minimizing the handling of OsO_4_ crystals and reducing toxic waste in laboratories. Nevertheless, our protocol can also be used with uranyl acetate, taking into consideration its limitations for CLEM. In addition, it can also be combined with genetically encoded markers, such as green fluorescent protein, quantum dots, or other selective dyes and antibodies. These refinements can expand the versatility of the workflow, offering a reliable tool for studying nanoscale biological structures.
